# Chloride Channel Mutations Leading to Congenital Myotonia

**DOI:** 10.7759/cureus.32649

**Published:** 2022-12-17

**Authors:** Amir Nik, Najmeh Ahangari, Paria Najarzadeh Torbati, Reza Boostani, Ehsan Ghayoor Karimiani

**Affiliations:** 1 Medical Genetics, Next Generation Genetic Polyclinic, Mashhad, IRN; 2 Medicine, Innovative Medical Research Center, Mashhad Medical Sciences, Islamic Azad University, Mashhad, IRN; 3 Neurology, Mashhad University of Medical Sciences, Mashhad, IRN

**Keywords:** molecular sequencing, genetic variant, muscle stiffness, congenital myotonia, chloride channel, creatine kinase, clcn1

## Abstract

Congenital myotonia is a non-dystrophic musculoskeletal disease that causes abnormal muscle relaxation. The prevalence of congenital disorders is notably high in Iran, emphasizing the importance of genetic assessment in suspicious cases. In this study, we aim to report cases with the chloride channel gene, CLCN1, mutations leading to significant morbidity. This case report study investigated four patients from four families with clinically defined congenital myotonia. Inclusion criteria were increased creatinine kinase (CK) and muscle stiffness. We collected data regarding family history, age of onset, and current therapeutic plan. All patients underwent skeletal muscle electromyography, cardiological evaluation, spirometry study, and hematochemistry assessment, including but not limited to muscle enzyme levels. Afterward, DNA was extracted from peripheral blood. Subsequently, whole exome sequencing (WES) and Sanger sequencing were done to detect and confirm variants, respectively. Age of onset ranged from 1 to 12 years in these patients, which are years apart from their first visit to the clinic. The warm-up phenomenon was present in all of them. A variant of uncertain clinical significance was found. We recommend that future research projects should study the efficiency of collaboration between clinicians, molecular geneticists, and other healthcare providers in order to find out about unclear variants as quickly as possible.

## Introduction

Congenital myotonia is a non-dystrophic musculoskeletal disease characterized by impairment of muscle relaxation after forceful contraction. It is due to mutations in the chloride channel gene, CLCN1, which is located on the seventh chromosome (7q34). This gene would translate into 988 amino acids with a molecular mass of 108626 Da [1]. This chloride channel (CIC-1) is a regulator of the electric excitability of skeletal muscle membrane playing a crucial role in both re-polarization and stabilization of the membrane potential [2].

Chloride channels play an important role in the regulation of cell volume, membrane potential stabilization, signal transduction, and transepithelial transport. CLCN1 loss-of-function mutations and gain-of-function mutations in the Sodium Channel Gene (SCN4A); both might lead to non-dystrophic myotonias [3].

Congenital myotonia, which is associated with mutations in CLCN1 has two different types: autosomal dominant inheritance type, which is called Thomsen's disease, and autosomal recessive inheritance type, which is known as Becker's type myotonia congenita (BTMC). These two diseases are distinguished by their inheritance mode and their clinical phenotypes, such as the age of onset, extent of myotonia, and genetic transmission pattern. The age of onset is early in childhood but usually older than in BTMC. Patients with BTMC display more severe clinical manifestations. Improving myotonia is noted with the repeated exercise of a given muscle, which is called the ‘warm-up phenomenon' [4].

A conservative estimate of the overall prevalence of inherited neuromuscular diseases among both sexes is around 1 in 3500 individuals who are expected to have disabling presentations in childhood or in later life [5]. The prevalence of the autosomal dominant and autosomal recessive types have been estimated to be 1:23,000 and 1:50,000, respectively. However, a large cohort study in the UK has proven otherwise, showing more prevalence in the recessive form [6]. In another UK sample including 665 patients with skeletal muscle channelopathies, 15 out of 104 different CLCN1 mutations accounted for 60% of all patients with myotonia congenita [7]. Studying the prevalence in Northern Scandinavia has been estimated at 1:10,000, in comparison to the worldwide prevalence being 1:100,000 [8]. An example would be Northern Finland. It most probably is the consequence of gene mutation enrichment, which has been proposed to be due to consanguineous marriage in this population [9].

Although acetylcholine, neostigmine, and potassium have been reported to deteriorate patients' condition in contrast to quinine, which reduced their disability. This would suggest that the site of the lesion is the neuromuscular junction [10]. On the other hand, it has been indicated that a muscular disorder is probable based on findings showing unusual muscular sensitivity to mechanical stimulus remaining unaffected by curare or degeneration of motor nerve endings [11].

Treatment in these patients can be problematic. Two sisters with BTMC demonstrated susceptibility to malignant hyperthermia (MH). General anesthetics like halothane or skeletal muscle relaxants like succinylcholine, which may be administered during surgical procedures, have been reported to cause MH in these patients. Hyperthermia, stiffness of skeletal muscles, hypotension, and arrhythmias might occur, requiring immediate emergency intervention [12]. There have been case reports discussing successful treatment in these patients. A 10-year-old girl with myotonia, "Herculean appearance," and electromyographic confirmation of myotonic discharges with dramatic response to carbamazepine [13]. Another case report studied the effect of dantrolene sodium on abolishing the myotonic phenomenon in Thomsen’s disease for a three-year-old patient. This medication, along with carbamazepine (CBZ), has been shown to decrease myotonic phenomenon [14].

In this study, we aim to report cases with CLCN1 mutations leading to significant morbidity.

## Case presentation

This case report study investigated four patients from four families with clinically deﬁned congenital myotonia. All patients who were referred to our laboratory were due to increased creatine kinase (CK) or muscle stiﬀness. On first inspection, information about family history, age of onset, and current therapeutic plan were collected. All patients underwent skeletal muscle electromyography, cardiological evaluation, spirometry study, and hematochemistry assessment including but not limited to muscle enzyme level. A consent form was obtained after providing detailed information from all patients or their legal guardians regarding genetic analysis and the use of their anonymized clinical data at the time of their first visit.

At first, DNA was extracted from the peripheral blood of the proband utilizing the GeneAll® Exgene trademark kit (GeneAll Biotechnology, Seoul, Korea), following the manufacturer’s protocol. Using Nanodrop (Thermo Fisher Scientific, Waltham, MA), DNA was analyzed using the levels of absorbance at different wavelengths. We performed whole exome sequencing (WES) with the Target Enrichment Preparation Kit by Agilent (Version V6, February 2018, Agilent Technologies, Santa Clara, California). Subsequently, products underwent 101 bp paired-end sequencing by utilizing the Illumina HiSeq 4000 system (Illumina, Inc., San Diego, CA). The generated FASTQ files were aligned to the human reference sequence (hg19) by Burrows-Wheeler Aligner (BWA), which resulted in the production of SAM files. Further SAM to BAM conversion, BAM file sorting, and removal of duplicate reads were carried out by Picard (http://picard.sourceforge.net). Local realignment and variant calling was done using the Genome Analysis Tool Kit (GATK; Broad Institute, Massachusetts Institute of Technology, Cambridge, MA) to create VCF files. Annotation was done using Annovar v322. The last step was Sanger sequencing to confirm the variant in patients and heterozygosity in healthy parents.

The age of onset ranged from 1 to 12 years in these patients. The warm-up phenomenon was present in 100% of them. Furthermore, they showed clinical myotonia in different parts of their bodies. We identified three previously reported mutations and one novel mutation in four patients.

Patient 1

A 33-year-old male who had difficulties opening his eyes when exposed to cold temperatures since he was three years old. His parents noticed an abnormal gait that could lead to falling while he was a toddler. Stiffness of tongue muscles, generalized transient weakness, and the warm-up phenomenon were noticed.

A homozygous variant in exon 15 of the CLCN1 gene (c.G1642A:p.E548K) was detected. This gene has previously been reported in association with BTMC (Phenotype MIM number: 255700). This mutation is absent in normal control databases used in the analysis pipeline. This variant is absent in the control groups used in Exome Sequencing Project, 1000 Genomes Project, and Exome Aggregation Consortium. Computational evidence supports a deleterious effect on the gene or gene product, including but not limited to conservation, evolutionary, and splicing impact. Based on this information, and in accordance with American College of Medical Genetics (ACMG) 2015 guidelines [15], we classify this as a Class 3 - variant of uncertain clinical significance.

Patient 2

A 12-year-old female was first observed being unable to lift heavy objects in her early teens. She complained of muscle stiffness and cramps, especially when climbing stairs, sitting, and running. Therefore, she warms up her muscles for better performance. The amount of serum CK was slightly elevated at 260 U/l (normal <200 U/). She had a brother who was three years younger with milder symptoms. Analysis showed homozygous changes in the fifth exon, with changes in DNA and protein showing c.C618A and p.Y206X, respectively.

Patient 3

This was a pair of 11-year-old twin females with the age of onset being two years old. They experienced muscular stiffness when starting to walk. Warm-up phenomenon and transient weakness were noticed. Climbing stairs and running were easier than sitting and standing. Laboratory tests showed an elevated level of serum CPK, which was 180 U/l (normal <170 U/). Genetic analysis showed homozygous mutations in the 9th exon with changes showing c.G1063A:p.G355R.

Patient 4

The proband was an eight-year-old female who reported pain, stiffness, and cramping in both hands and shoulder accompanied by severe respiratory problems since she was four years old. Therefore, she needed supplemental oxygen for better breathing.

The twenty-third exon demonstrated one heterozygous mutation c.C2926T:p.R976X on the CLCN1 gene. This mutation has been reported in patients with congenital myotonia. The frequency of this mutation in the normal population is very low.

## Discussion

There have been programs in place to screen for reproductive carriers of recessive diseases including but not limited to Tay-Sachs disease, which started in the 1970s [16]. These programs can enhance a couple’s informed decision-making when trying to conceive by providing them with information regarding the risk of autosomal or X-linked recessive disorders [17]. There are federally funded investigations currently in place in order to research population-based reproductive carrier screening by finding the frequency, morbidity, and mortality of congenital myopathies [18]. The drive for treatment is becoming more important since novel techniques, including but not limited to exon skipping, RNA interference, and adeno-associated virus (AAV)-mediated gene replacement, have led to a higher rate of diagnosis [19].

In this study, we found CLCN1 mutations indicating probable relation to non-dystrophic myotonia. This phenomenon could be related to Iran’s high rate of consanguineous marriage of 38.6% with the highest among first cousins [20]. The frequency of congenital disorders in Iran has been reported as 38.3 per 1,000 people, which is notably high. This includes single-gene disorders with a prevalence of 16.4 per 1,000 individuals [21].

The findings of this study have to be seen in light of some limitations. Due to time constraints, the sample size is not enough to conduct a statistical analysis. Obtaining gene sequencing results were time-consuming due to limited resources and the voluntary nature of the research. In order to expedite diagnostic yield, clinical exam sequencing (CES), which analyzes fewer genes in comparison to whole genome sequencing, has been proposed to be a cost-effective, powerful, first-line diagnostic tool in establishing the molecular diagnosis of adult patients with variable symptoms, including patients with skeletal muscle channelopathies. Other non-genetic diagnostic methods and their correlation with severity including but not limited to cardiac conduction studies should be assessed in the future to underscore more severe cases.

## Conclusions

We conclude the age of onset to be mainly during early childhood, which is years apart from their first visit to the clinic. This issue might be due to limited resources available for genetic counseling and/or the low socioeconomic status of these patients. Moreover, the lack of efficient communication to report and refer cases with undiagnosed conditions for further genetic counseling could be another factor playing a role in the proper management of these disorders.

We believe that healthcare policies should be designed in a way to enhance the delivery of preventative care in populations with a higher risk for congenital abnormalities. We recommend that future research projects should study the efficiency of collaboration between clinicians, molecular geneticists, and other healthcare providers in order to find out about unclear variants as quickly as possible.

## References

[1] (2022). VCV000252462.12. ClinVar. NCBI. https://www.ncbi.nlm.nih.gov/clinvar/variation/252462/.

[2] Jentsch TJ, Pusch M (2018). CLC chloride channels and transporters: structure, function, physiology, and disease. Physiol Rev.

[3] Stunnenberg BC, LoRusso S, Arnold WD (2020). Guidelines on clinical presentation and management of nondystrophic myotonias. Muscle Nerve.

[4] Özgün N, Taşlıdere H (2020). Congenital myotonia: a review of twenty cases and a new splice-site mutation in the CLCN1 gene. Turk J Pediatr.

[5] Emery AE (1991). Population frequencies of inherited neuromuscular diseases—a world survey. Neuromuscul Disord.

[6] Harper PS (1975). Congenital myotonic dystrophy in Britain. I. Clinical aspects. Arch Dis Child.

[7] Horga A, Raja Rayan DL, Matthews E (2013). Prevalence study of genetically defined skeletal muscle channelopathies in England. Neurology.

[8] Müller KI, Ghelue MV, Lund I, Jonsrud C, Arntzen KA (2021). The prevalence of hereditary neuromuscular disorders in Northern Norway. Brain Behav.

[9] Baumann P, Myllylä VV, Leisti J (1998). Myotonia congenita in northern Finland: an epidemiological and genetic study. J Med Genet.

[10] Hoppe K, Chaiklieng S, Lehmann-Horn F, Jurkat-Rott K, Wearing S, Klingler W (2020). Preclinical pharmacological in vitro investigations on low chloride conductance myotonia: effects of potassium regulation. Pflugers Arch.

[11] Georgesco M, Salerno A (2000). Spontaneous activity in electromyography: mechanisms and practical interest. Neurophysiol Clin.

[12] (2022). Myotonia congenita. NORD (National Organization for Rare Disorders). https://rarediseases.org/rare-diseases/myotonia-congenita/.

[13] Savitha MR, Krishnamurthy B, Hyderi A, Farhan-Ul-Haque Farhan-Ul-Haque, Ramachandra NB (2006). Myotonia congenita — a successful response to carbamazepine. Indian J Pediatr.

[14] Ohtaki E, Komori H, Yamaguchi Y, Matsuishi T (1991). Successful dantrolene sodium treatment of a patient with myotonia congenita (Thomsen's disease). Acta Paediatr Jpn.

[15] Richards S, Aziz N, Bale S (2015). Standards and guidelines for the interpretation of sequence variants: a joint consensus recommendation of the American College of Medical Genetics and Genomics and the Association for Molecular Pathology. Genet Med.

[16] Kaback M, Lim-Steele J, Dabholkar D (1993). Tay-Sachs disease— carrier screening, prenatal diagnosis, and the molecular era. An international perspective, 1970 to 1993. JAMA.

[17] Henneman L, Borry P, Chokoshvili D (2016). Responsible implementation of expanded carrier screening. Eur J Hum Genet.

[18] Belcher A, Mangelsdorf M, McDonald F, Curtis C, Waddell N, Hussey K (2019). What does Australia's investment in genomics mean for public health?. Aust N Z J Public Health.

[19] Ravenscroft G, Bryson-Richardson RJ, Nowak KJ, Laing NG (2018). Recent advances in understanding congenital myopathies. F1000Res.

[20] Saadat M, Ansari-Lari M, Farhud DD (2004). Consanguineous marriage in Iran. Ann Hum Biol.

[21] Behnam B, Zakeri M (2019). Genetics and genomic medicine in Iran. Mol Genet Genomic Med.

